# Efficient Genome Editing in Multiple Salmonid Cell Lines Using Ribonucleoprotein Complexes

**DOI:** 10.1007/s10126-020-09995-y

**Published:** 2020-09-18

**Authors:** Remi L. Gratacap, Ye Hwa Jin, Marina Mantsopoulou, Ross D. Houston

**Affiliations:** The Roslin Institute and Royal (Dick) School of Veterinary Studies, University of Edinburgh, Midlothian, EH25 9RG UK

**Keywords:** CRISPR, Salmonid, Cell line, Ribonucleoprotein, Genome editing, Disease resistance

## Abstract

Infectious and parasitic diseases have major negative economic and animal welfare impacts on aquaculture of salmonid species. Improved knowledge of the functional basis of host response and genetic resistance to these diseases is key to developing preventative and treatment options. Cell lines provide valuable models to study infectious diseases in salmonids, and genome editing using CRISPR/Cas systems provides an exciting avenue to evaluate the function of specific genes in those systems. While CRISPR/Cas editing has been successfully performed in a Chinook salmon cell line (CHSE-214), there are no reports to date of editing of cell lines derived from the most commercially relevant salmonid species Atlantic salmon and rainbow trout, which are difficult to transduce and therefore edit using lentivirus-mediated methods. In the current study, a method of genome editing of salmonid cell lines using ribonucleoprotein (RNP) complexes was optimised and tested in the most commonly used salmonid fish cell lines: Atlantic salmon (SHK-1 and ASK cell lines), rainbow trout (RTG-2) and Chinook salmon (CHSE-214). Electroporation of RNP based on either Cas9 or Cas12a was efficient at targeted editing of all the tested lines (typically > 90% cells edited), and the choice of enzyme expands the number of potential target sites for editing within the genomes of these species. These optimised protocols will facilitate functional genetic studies in salmonid cell lines, which are widely used as model systems for infectious diseases in aquaculture.

**Electronic supplementary material:**

The online version of this article (10.1007/s10126-020-09995-y) contains supplementary material, which is available to authorized users.

## Introduction

Salmonid fish are amongst the highest value aquaculture species globally, together worth in excess of $22Bn in 2017 (FAO 2019). However, infectious disease outbreaks are a continuous threat to sustainable production and future expansion to meet global demands for these fish. Therefore, development of vaccines and therapeutics is an important goal, and selective breeding for improved host resistance has major potential to help tackle several diseases (Yáñez et al. 2014). Genomic selection has also been applied to enhance the rate of genetic gain for disease resistance traits in breeding programmes (Houston 2017; Zenger et al. 2019; Houston et al. 2020), and genome editing approaches may offer further step-improvements in the future (Gratacap et al. 2019). However, research into the functional mechanisms underlying host response to salmonid pathogens, and host genetic variation in resistance is important to support development of these potential solutions.

Genome editing using CRISPR/Cas systems is a valuable research tool because it allows targeted changes to genomes of species of interest. Therefore, CRISPR/Cas editing can be applied to test the functional role of a particular gene or variant in a trait of interest, such as resistance to infection (Staller et al. 2019). This can be achieved by editing the target species’ genome at a location that will result in knockout of the target gene, or by introducing domains which will activate or repress its expression (Gilbert et al. 2014; Doudna and Charpentier 2014). CRISPR/Cas9 has successfully been applied in vivo to edit the genome of Atlantic salmon (Edvardsen et al. 2014) and rainbow trout (Cleveland et al. 2018), including to create a sterile salmon by knockout of the *dnd* gene (Wargelius et al. 2016). In addition to understanding gene function, genome editing holds significant potential to be applied in commercial aquaculture to tackle major production barriers (Gratacap et al. 2019).

The use of cell lines for research into salmonid pathogens and host response has been well-established, and engineering of those cell lines holds substantial promise for advancing fish health research (Collet et al. 2018). However, genome editing of salmonid cell lines remains in its infancy, with the first report of successful CRISPR/Cas editing being in the Chinook salmon (*Oncorhynchus tshawytscha*) cell line (CHSE-EC, derived from CHSE-214), which was engineered to stably express Cas9 and EGFP (Dehler et al. 2016). This line has subsequently been applied to develop a clonal STAT2 knockout line to study the role of this gene in viral response (Dehler et al. 2019), and as a proof-of-principle to demonstrate that transduction of lentivirus facilitates high-efficiency editing (Gratacap et al. 2020). Additionally, Escobar-Aguirre et al. (2019) reported delivery and expression of a gRNA, Cas9, and an mCherry reporter gene in CHSE-214 using a plasmid construct. However, other salmonid fish cell lines are considered difficult to transfect and to develop clonal lines (Collet et al. 2018), making analogous approaches in existing Atlantic salmon and rainbow trout cell lines challenging. Indeed lentivirus transduction using the approach of Gratacap et al. (2020) resulted in a very low success rate in the Atlantic salmon SHK-1 cell line (< 1% of cells successfully transduced; data not shown). CRISPR/Cas ribonucleoprotein (RNP) complexes have potential for editing of fish cell lines, as demonstrated with efficiency of up to 62% in medaka (*Oryzias latipes*) (Liu et al. 2018). Furthermore, Cas12a editing has been successfully applied in mammalian cells, *Xenopus* and zebrafish (*Danio rerio*), including using RNP systems (Moreno-Mateos et al. 2017; Liu et al. 2019), and significantly expands the number of ‘editable’ sites in the target species’ genomes. The efficiency of Cas9 or Cas12a editing using RNP in salmonid cell lines is unknown, and these approaches may help overcome the aforementioned challenges to cell line editing in cell lines of Atlantic salmon and rainbow trout, two of the world’s most important aquaculture species.

In the current study, a simple and reproducible method of editing multiple salmonid fish cell lines using electroporation of Cas9 RNP complexes is presented. The method was tested and optimised, resulting in very efficient editing of all the commonly used salmonid cell lines tested, specifically Atlantic salmon (ASK and SHK-1), rainbow trout (RTG-2) and Chinook salmon (CHSE-214). Additionally, electroporation of Cas12a RNP which uses a different protospacer adjacent motif (PAM) of 5’TTTV led to high genome editing (although less than Cas9), expanding the number of potential target editing sites in these species’ genomes.

## Materials and Methods

### Cell Lines

The cell lines used in this study were as follows: (i) salmon head kidney 1 (SHK-1), an immortalised cell line from Atlantic salmon (*Salmo salar*) obtained from the European Collection of Authenticated Cell Cultures (ECACC) (97111106); (ii) Atlantic salmon kidney (ASK), an immortalised cell line from Atlantic salmon (*S. salar*) obtained from American Type Culture Collection (ATCC; CRL-2747); (iii) rainbow trout gonad (RTG-2), an immortalised cell line from rainbow trout (*O. mykiss*) obtained from ECACC (90102529); and (iv) Chinook salmon embryo 214 (CHSE-214), an immortalised cell line from Chinook salmon (*O. tshawytscha*) obtained from ECACC (91041114). All cells were grown as a monolayer in L15 media (Sigma-Aldrich, St. Louis, USA) supplemented with heat-inactivated foetal bovine serum (FBS) (Gibco, Waltham, USA) (SHK-1, 5%; RTG-2, CHSE-214 and ASK, 10%), 40 μM β-mercaptoethanol (Gibco) for SHK-1, 100 units/mL penicillin and 100 μg/mL streptomycin (Gibco). All cells were cultured in an incubator at 22 ± 1 °C without CO_2_. SHK-1 was split 1:2 at 80% confluency with  conditioned media and the rest of cell lines were split 1:3–1:4 with fresh media.

SHK-1 cells were used for the initial optimisation of RNP editing, as described below. As part of this process, an SHK-1 line with a GFP transgene was created to allow testing of gRNA targeting knockout of this transgene causing loss of fluorescence. This cell line (SHK-FuGFP) was generated by transfecting a CMV-GFP_Puromycin construct (Addgene 45561, a gift from Michael McVoy) in SHK-1 cells with Fugene HD transfection reagent (Promega, Madison, USA). To achieve this, SHK-1 cells were plated in a 24-well plate at 40,000 cells per well and incubated overnight at 22 °C. Media was replaced with 500 μL of L15 (10% FBS, no antibiotics) containing 0.5 μg plasmid and 1.5 μL of FugeneHD (ratio Fugene:DNA 3:1, according to manufacturer’s instructions). After 7 days, cells were selected with puromycin at a concentration of 1 μg/mL for a period of 4 weeks.

### Optimisation of Cas9 RNP Transfection and Editing

To test and optimise the Cas9 RNP platform, an intergenic region of the Atlantic salmon genome (GenBank accession NC_027325.1 ssa26; 15004350–15004900) was targeted with a gRNA via transfection of the SHK-1 cell line. This was followed by validation of the optimised conditions by EGFP knockout in the SHK-FuGFP cell line and knockout of coding region of *slc45a2* (Gene ID: 106563596).

The crRNAs were designed with CRISPOR (http://crispor.tefor.net/) and the CRISPR Design Tool (Synthego Inc., Menlo Park, USA), and crRNAs and tracrRNAs were ordered from IDT (details of all gRNA are given in Table 1). The RNP complexes were assembled as follows: crRNA and tracrRNA were resuspended in nuclease-free water at 100 μM, aliquoted and frozen at − 80 °C. One microlitre of crRNA and 1 μL of tracrRNA were mixed and incubated at 95 °C for 5 min. The mixture was cooled to room temperature, and 2 μL of 20 μM Cas9 (NEB, Ipswich, USA) was added (final concentration of 10 μM of Cas9 and 25 μM of gRNA). The complexes were incubated at room temperature for 15 min and kept on ice until use.

**Table 1:** Guide RNA sequences and primers used for amplification and sequencing of target genomic regions

CRISPR effector, target	Guide sequences (5′ – 3′)	Primers (5′ – 3′)	Size (bp)	Tm (°C)
spCas9, intergenic	TCCCAACGTGCTATCCATCT	F1: GACACTGTGGTGAATTTGCTATT R1: CCCAGTAGTAGCTTGAAAGAGG	479	61.5
spCas9, EGFP	GAGCTGGACGGCGACGTAAA	F1: CGCAAATGGGCGGTAGGCGTG R1: GTCTTGTAGTTGCCGTCGTC	471	63
spCas9, *slc45a2* (exon 6)	AGCCCCTTCAGACCGATGTA	F1: CAATCACAGGTGGGAAAAGGGC R1: GAGGGTACTGACCTCCTCCTCA	528	66
spCas9, *slc45a2* (exon 1)	GGACTGTAGGGAGTCTACGA	F1: GCCATTGACAAGCGGGCTGA R1: TGCGAGGATGTAGGGCCTCC	469	67
AsCas12a, *slc45a2* (exon 6)	GTCTGGGCACCAGTCTTATCG	F1: CAATCACAGGTGGGAAAAGGGC R1: GAGGGTACTGACCTCCTCCTCA	528	66
		F2*: TGACCGGAACACAGCAGAAGGGT R2*: ACAGGTGGTGGATGAGGTTCGCA	529	67

*F2/R2 primer pair is for *O. tshawytsha* (CHSE-214) and the rest of the *slc45a2* primer pairs are for both *S. salar* (SHK-1 and ASK) and *O. mykiss* (RTG-2)

The first optimisation step involved varying the concentration of the Cas9-gRNA RNP complex, with the starting point being electroporation conditions that have previously been successful in plasmid transfection of SHK-1 (data not shown). To achieve this, different concentrations of RNP were diluted in OptiMEM reduced serum media (Gibco) (final volume 4 μL) and mixed with 10 μL of SHK-1 cells at 10^7^ cells/mL in OptiMEM (final concentrations range from 0.0875 to 2.8 μM Cas9 RNP). After 5-min incubation at room temperature, the cells plus the RNP were electroporated with the Neon system (Invitrogen, Carlsbad, USA) according to the manufacturer’s instructions but with OptiMEM instead of Neon R Buffer (Invitrogen). The mixture was electroporated using 10 μL tips and dispensed in 1 mL of fresh media in a 24-well plate. One hundred microlitres of the suspension was transferred to a 96-well plate (10^4^ cells) for genomic DNA isolation or cell culture. The cells were incubated overnight at room temperature, and the media changed to 1 mL of fresh media. Once the cells reached confluency (in 24-well plates), they were resuspended in media (using trypsin) and divided 1:2 (adding 33% conditioned media for the SHK-1 cells). Cells were kept in 96- or 24-well plates for gDNA isolation at 1, 2, 4, 7, and 14 days post treatment (dpt) or expanded to 6-well plates once they reached confluency for measurement of fluorescence using flow cytometry at 14 dpt.

### Cell Survival

In addition to assessing the transfection and editing efficiency of the SHK-1 cells, the cell viability was tested in parallel for each of the setting used for the optimisation protocol using CellTiter-Glo 2.0 (Invitrogen) and cells with Cas9 RNP complex but not electroporated as controls. In brief, following electroporation, 100 μL of the cells in the 24-well plate were transferred to a 96-well plate and incubated for 48 h. Surviving cells still attached to the bottom of the plate were rinsed once in PBS and 120 μL of CellTiter-Glo solution (diluted 1:10 in PBS) was added to each well. The plate was incubated in the dark for 30 min on a plate rocker at room temperature and 100 μL of the solution was transferred to a flat bottom white wall 96-well plate (Greiner Bio-One, Austria). The luminescence was measured using a Cytation3 imaging reader and the Gen5 software V3.03 (BioTek, Winooski, USA).

### Validation of Optimised Cas9 RNP Editing by GFP Knockout

A second test of RNP editing was performed in the SHK-fuGFP cells by using the optimised settings to transfect an RNP complex with a gRNA targeting knockout of the GFP transgene. Following the transfection, the loss of GFP was measured by flow cytometry. To achieve this, the cells were trypsinised and resuspended in PBS. The cells were kept on ice and flow cytometry was performed using a Fortessa-X20 (BD Biosciences, San Jose, USA). Single-cell events were gated, and the percentage of GFP-positive cells and the intensity of GFP fluorescence from each cell was measured.

### Assessing the Efficiency and Nature of the Edits Using Sanger Sequencing

Testing of editing efficiency was performed by isolation of genomic DNA followed by PCR amplicon Sanger sequencing. Genomic DNA (gDNA) was extracted with QuickExtract buffer (Lucigen, Middleton, USA) by adding 30 μL to a well of a 96-well plate and incubating for 5 min. The samples were then processed according to the manufacturer’s instructions (65 °C for 15 min and 98 °C for 2 min). PCR was performed with 50 μL reactions using NEB Q5 and 1 μL of the gDNA with 33 cycles of amplification at optimal annealing temperature (Table 1). Five microlitres of the PCR product was run on a 1.5% agarose gel to verify correct amplification. Amplified sequence was purified with AmPURE XP magnetic beads (Agencourt, Beverly, USA) according to the manufacturer’s instructions (using 1:1 ratio) and sent to GATC/Eurofins (Germany) for Sanger sequencing. Analysis of the chromatograms (based on .abi files) was used to assess the editing efficiency and nature of the induced edits using the Inference of CRISPR Edits (ICE, Synthego Inc) software for Cas9 or TIDE software (Brinkman et al. 2014) for Cas12a to determine the editing efficiency (% of cells containing putative indels).

### Testing of RNP Editing in Other Salmon Cell Lines

Following optimising of the electroporation and incubation settings for the SHK-1 cells described above, similar protocols were tested in the other three cell lines by targeting the *slc45a2* gene. Several combinations of different electroporation settings (1200–1600 V, 10–40 ms, 1–3 pulses) and cell resuspension buffers (Neon R buffer and OptiMEM) were tested to achieve highest transfection rate using tracrRNA–ATTO550 (IDT, Coralville, USA) by detecting ATTO550-positive cell population using flow cytometry at 24 h post electroporation. The best transfection result (99.9–100%) was obtained with 1400 V 20 ms 1 pulse for RTG-2 and 1600 V 10 ms 3 pulses for CHSE-214 and ASK with OptiMEM as a resuspension buffer (data not shown).

The Cas9 RNP complex was assembled as described above and Cas12a RNP was formed by adding 31.2 pmol of AsCas12a (IDT) and 50 pmol of crRNA (IDT) per 10^5^ cells. The complexes were incubated at room temperature for 15 min and kept on ice until use. The final concentration of 1 μM of Cas9 RNP and 2.6 μM of AsCas12a RNP were tested with the optimised electroporation settings for each cell line. At 7 dpt, the editing efficiency and the nature of the induced edits were assessed by Sanger sequencing and ICE software for Cas9 or TIDE software for Cas12a as described above.

## Results

### Electroporation of Cas9-gRNA Complex Leads to Efficient Editing

**Fig. 1:**
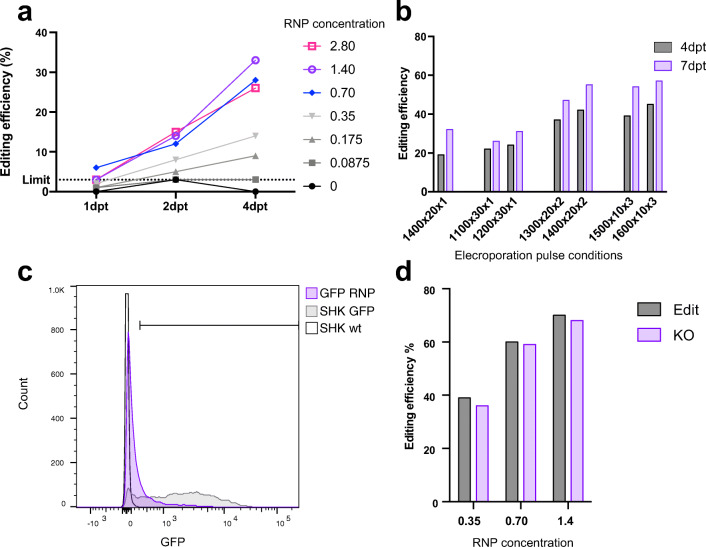
Efficient editing of Atlantic salmon cell line by electroporation of Cas9 RNP. **a**, **b** Optimisation of genome editing in SHK-1 cells targeting an intergenic region. SHK-1 cells were electroporated (1300 V, 30 ms and 1 pulse) with different concentrations of Cas9 RNP (μM) and gDNA isolated at different timepoints after electroporation (days post transfection, dpt). **b** Using the optimal concentration (1.4 μM) of RNP, different electroporation settings were evaluated [voltage (V) × pulse duration (ms) × number of pulses] and the sampling time increased to 4 and 7 dpt. **c**, **d** Efficient knock out of GFP in SHK-GFP cells. SHK-fuGFP was electroporated with optimised settings and gRNA targeting GFP transgene. After 14 days, fluorescence was measured using flow cytometry (**c**), and editing efficiency was also assessed by Sanger sequencing (**d**). All genome editing efficiency generated using ICE (Synthego Inc) deconvolution of Sanger sequencing chromatogram. ‘Edit’ refers to the estimated percentage of edited cells, while ‘KO’ refers to the estimated percentage of cells which contain edits expected to result in GFP knockout

To test and optimise the CRISPR/Cas9 genome editing platform using RNP, an intergenic region of the Atlantic salmon genome was targeted in the SHK-1 cell line. The first optimisation step involved varying the concentration of the Cas9 RNP complex, with set electroporation conditions (previously optimised for plasmid transfection of SHK-1: 1300 V, 30 ms and 1 pulse). The editing efficiency increased with increasing concentration of RNP up to 1.4 μM, but plateaued at higher concentrations (Fig. 1a). This RNP concentration was then used for optimisation of electroporation settings. Both cell survival and editing efficiency (Fig. S1a and Fig. S1b) were assayed. Using three pulses of 1600 V for 10 ms resulted in the highest editing rate of 42% at 4 days posttreatment (dpt, Fig.1b), and also the highest cell survival (113% survival compared to control, Fig. S1b). To assess whether editing was still occurring after 4 days, samples of the SHK-1 cells were taken for Sanger sequencing at 7 and 14 dpt. The proportion of edited cells was higher after 7 days than 4 days, but did not increase afterwards (Fig. 1b and Fig. S1c). While most of the experimental conditions in the optimisation experiment were based on a single sample, the resulting optimised RNP editing protocol for SHK-1 cells led to reproducible editing with 56 and 57% of the cells edited using electroporation of 1.4 μM RNP with three 1600 V pulses of 10 ms, after 7 days in two independent experiments (Fig. S1d). It is worth noting that the pattern of edits (+ 1, − 1 and − 6 bp edits) generated by this gRNA:Cas9 complex is reproducible as seen in Fig. S1e.

Further validation of the Cas9 RNP electroporation platform was performed using an EGFP knockout system in SHK-1 cells. An SHK-1 cell line with constitutive EGFP expression was created, and an RNP complex targeting EGFP was designed. Using the optimised electroporation and RNP concentration established above, there was approximately 75% loss of GFP as measured by flow cytometry (Fig. 1c), with an estimated 68% editing efficiency by Sanger sequencing (Fig. 1d).

### Optimised Protocol Translates to Efficient Editing in Multiple Cell Lines Using Cas9 and Cas12a Enzymes

**Fig. 2:**
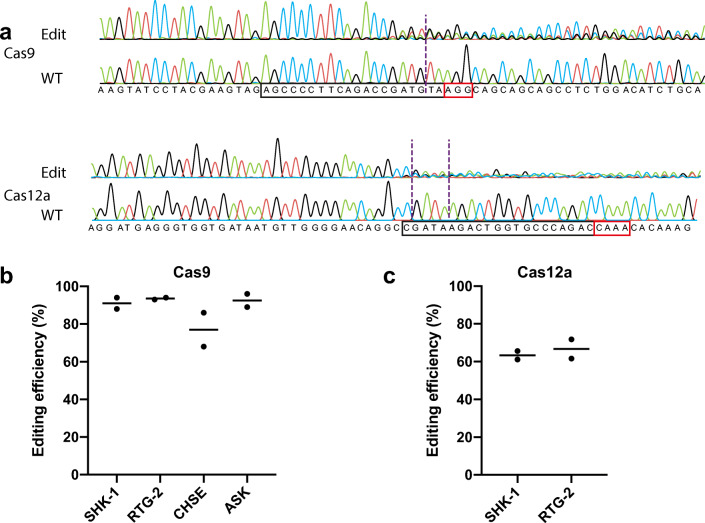
Efficient editing of salmonid cell lines with different Cas proteins. **a** Representative chromatogram of the Sanger sequencing for the target region of the *slc45a2* gene in SHK-1 cells, either wild-type (WT) or edited with Cas9 (top) and Cas12a (bottom) RNP. The binding regions are boxed in black and PAM sequence is in red. The nuclease cut positions are indicated by dashed lines. **b** Editing of *slc45a2* gene in SHK-1, ASK, RTG-2 and CHSE-214 using Cas9 RNP. **c** Editing of *slc45a2* gene in SHK-1 and RTG-2 with Cas12a RNP. Two independent experiments (with median) are represented. Editing efficiency was estimated with ICE and TIDE analyses for Cas9 and Cas12a RNP, respectively

To evaluate the potential of RNP editing in the most commonly used salmonid cell lines, the optimised settings established were used to target a coding region of *slc45a2* which is involved in pigmentation and has been successfully knocked out using CRISPR/Cas9 in Atlantic salmon (Edvardsen et al. 2014). The cell lines targeted were SHK-1 and ASK (*S. salar*), RTG-2 (*O. mykiss*) and CHSE-214 (*O. tshawytsha*). Electroporation of Cas9 RNP targeting *slc45a2* resulted in over 90% of cells edited in SHK-1, RTG-2 and ASK and over 70% edited in CHSE-214 (Fig. 2a and b), which was consistent across two independent experiments. It is worth noting that the same gRNA was used in all cell lines but there was a 1 bp mismatch with the homologous target region of Chinook salmon (CHSE-214), which may explain the lower editing observed.

The nature of the edits in the target region was assessed using Sanger sequencing of PCR amplicons followed by analysis using the ICE software (as described above). This method of quantifying editing efficiency and the nature of the edits using Sanger sequencing has previously been shown to have a high concordance with results from next generation sequencing analyses (Brinkman et al. 2018; Hsiau et al. 2020). In both experiment replicates, the majority of SHK-1 cells edited by Cas9 contained a 1 bp deletion, whereas the same gRNA primarily resulted in a 1 bp insertion in RTG-2 cells (Fig. S2b). While the pattern of indels observed when using the same gRNA varied between cell lines, single base pair insertions or deletions were predominant (Fig. S2b).

The Cas12a RNP targeted the same genomic region as for Cas9 RNP (*slc45a2* Exon 6 Ssa01:117877060–117877079 for Cas9 and Ssa01:117877013–117877033 bp for Cas12a), and Cas12a also resulted in high editing efficiency in both Atlantic salmon (SHK-1, 63%) and rainbow trout (RTG-2, 67%) cell lines (Fig. 2c), although the editing rates were notably lower than when using the Cas9 enzyme.

## Discussion

The current study presents an optimised method for genome editing in the most commonly used salmonid cell lines using electroporation of Cas9 or Cas12a RNP. The method can be used to edit over 90% of cells in a mixed cell line population with Cas9, and over 60% with Cas12a; this is generally a higher editing efficiency than reported in medaka fish cell lines using Cas9 RNP (62% editing, Liu et al. 2018). The optimised RNP method circumvents several challenges to genetic engineering of many fish cell lines due to their slow growth, poor transfection/transduction efficiency, and difficulty to obtain clonal lines (Collet et al. 2018).

In the current study, Cas9 RNP electroporation was optimised by testing a wide range of voltage (850–1600 V), time (10–40 ms) and number of pulse (1–3) variables, and the most effective electroporation setting for SHK-1 was shown to be with 1600 V 10 ms 3 pulses. Interestingly, these settings were also optimal for CHSE-214 and ASK, while the optimal electroporation setting for RTG-2 was 1400 V 20 ms 1 pulse. This is the first report of electroporation of protein: RNA complex in salmonid cells. The differences in optimal electroporation settings compared with previous studies using plasmide may be explained by the difference in cargo or the use of OptiMEM instead of buffer R for the electroporation (Ojima et al. 1999; Chi et al. 2012; Marivin et al. 2015). These settings were also optimal for electroporation and editing using Cas12a RNP in both Atlantic salmon and rainbow trout cell lines. The use of Cas12a as well as Cas9 increases the range of targetable sequences for editing (5’NGG and 5’TTTV) in salmonid species’ genomes.

Interestingly, editing efficiency in the SHK-1 cell line was higher at 7 dpt than 4 dpt. This implies that editing is occurring more slowly than in mammalian systems (Kim et al. 2014) and highlights that the Cas9 protein is still active for over a week in the experimental conditions described (cells incubated at room temperature). It is also noteworthy that both gRNAs targeting *slc45a2* were more efficient than the gRNA targeting the intergenic region. This highlights that the efficacy of the system will vary across different target genomic regions, which may be due to differences in chromatin accessibility (Uusi-Mäkelä et al. 2018). Therefore, certain genes and genomic locations may not be amenable to highly efficient editing using this approach. This problem also applies to lentivirus and plasmid delivery systems, but low efficiency editing in these systems can be combined with selection using antibiotics or fluorescence to enrich for edited cells, which is not possible using the RNP editing system described herein. Ultimately, single-cell cloning might be required to achieve a high level of editing (possibly 100% edited clones) for sites with lower editing efficiency using the current system. However, single cell cloning has not been successful in SHK-1 cells, and the process is likely to take several months in the other salmonid cell lines due to their slow-growing nature.

By utilising Cas RNP complexes, the method presented here allows rapid and efficient editing of salmonid cell lines. From design to experimental testing of the edits, the protocol takes just over 2 weeks. This is approximately half the time required for lentivirus or plasmid delivery as the constructs have to be generated and sequenced before delivery and then enrichment applied. Additionally, lentivirus and plasmid approaches require investigation and optimisation of the promoter (Ruiz et al. 2008; Martinez-Lopez et al. 2013) and selection marker (Schiøtz et al. 2011) choices for in each cell line since little information is yet available for most fish cell lines. Finally, the very high editing rate (typically > 90%) obtained with the method described herein also circumvents the need for enrichment of the edited population, allows direct testing of the cells for the phenotype of interest.

The salmonid fish cell lines used in the current study are widely used model systems to understand genetics and immunology of commercially and environmentally important fish species. The ability to perform targeted gene knockout will allow for assessment of candidate genes involved in genetic resistance to disease, for example. The in vitro system could also act as a testbed for gRNAs efficiency prior to their use in vivo. Given the scientific and commercial interest in salmonid fish species, and that all the cells line tested could be efficiently edited, this technique is likely to form a useful component of the toolbox for functional genetics and immunology research in fish.

## Electronic supplementary material

ESM 1 (DOCX 212 kb)
