# Supplementary material for: Efficient Genome Editing in Multiple Salmonid Cell Lines Using Ribonucleoprotein Complexes
Source: Mar Biotechnol (NY). 2020 Sep 18;22(5):717–24. doi: 10.1007/s10126-020-09995-y (PMC7520412; doi:10.1007/s10126-020-09995-y)
Supplement: Supplementary file 1 — (DOCX 212 kb) [file 10126_2020_9995_MOESM1_ESM.docx]

**Supplementary Figure S1.** SHK-1 cells were electroporated with 1.4 µM Cas9:gRNA RNP and transferred to 2 separate 96-well plates. (a) After 48h, cell survival (plate1) was calculated using CellTiter Glo 2.0. (b) genomic DNA (plate 2) was extracted at 7 dpt, and the target sequence amplified by PCR and editing efficiency estimated using Sanger sequencing. (c) Using 1.4 µM Cas9 RNP, the editing efficiency after 7 and 14 dpt was estimated for different electroporation settings. (d) Editing efficiency of electroporation with 1.4 µM Cas9 RNP after 7 days was similar between two independent experiments. (e) All the sequencing data, obtained from ICE analysis of Sanger sequencing of the intergenic target region from optimisation experiments (n=55) were pooled and plotted according to edit pattern.

#

# **Supplementary Figure S2.** (A) Editing of *slc45a2* gene in SHK-1 with Cas9 RNP with two gRNA targeting different exons (Exon 1 and Exon 6). (B) Detail of the Indel frequency estimated for *slc45a2* with Cas9 in different cell lines (refers to Fig 2B). Indels with frequencies over 2 % are represented. Two independent experiments (with median) are represented. Editing efficiency was estimated using ICE analysis.
